# Supplementary material for: Social learning mechanisms shape transmission pathways through replicate local social networks of wild birds
Source: eLife. 2023 May 2;12:e85703. doi: 10.7554/eLife.85703 (PMC10154030; doi:10.7554/eLife.85703)
Supplement: Supplementary file 1. — (a) Correlation coefficients between the three individual network metrics. Shown are the correlation coefficient between the network metrics weighted clustering coefficient, degree, and betweenness. (b) Effects of network size on the mean correlation coefficient. Shown are the effects of network size on the mean correlation coefficient for weighted clustering coefficient, degree, and betweenness for each of the four social learning models. We report the estimate, the standard error (SE), test statistic (t), and p values. [file elife-85703-supp1.docx]

**Supplementary tables**

**Table a. Correlation coefficients between the three individual network metrics.** Shown are the correlation coefficient between the network metrics weighted clustering coefficient, degree and betweenness.

|  | **Weighted degree** | **Weighted clustering coefficient** |
| --- | --- | --- |
| **Weighted clustering coefficient** | -0.31 |  |
| **Weighted betweenness** | 0.39 | -0.34 |

**Table b.** **Effects of network size on the mean correlation coefficient.** Shown are the effects of network size on the mean correlation coefficient for weighted clustering coefficient, degree and betweenness for each of the four social learning models. We report the estimate, the standard error (SE), test statistic (t) and P values.

| **Dependent variable** | **Model** | **Fixed effects** | **Estimate** | **SE** | **t** | **P** |
| --- | --- | --- | --- | --- | --- | --- |
| ***Mean correlation coefficient for weighted clustering coefficient*** | ***Simple*** | Intercept | 0.05 | 0.01 | 3.83 | 0.01 |
|  |  | Network size | 0.002 | 0.0004 | 6.52 | <0.001 |
|  | ***Threshold*** | Intercept | -0.004 | 0.002 | -1.9 | 0.07 |
|  |  | Network size | 0.0004 | 0.0001 | 4.57 | <0.001 |
|  | ***Proportion*** | Intercept | 0.001 | 0.003 | 0.37 | 0.71 |
|  |  | Network size | 0.0001 | 0.0001 | 1.29 | 0.2 |
|  | ***Conformity*** | Intercept | -0.01 | 0.003 | -3.05 | 0.003 |
|  |  | Network size | 0.0001 | 0.0001 | 1.03 | 0.3 |
| ***Mean correlation coefficient for weighted degree*** | ***Simple*** | Intercept | -0.24 | 0.01 | -20.68 | <0.001 |
|  |  | Network size | -0.002 | 0.0002 | -8.53 | <0.001 |
|  | ***Threshold*** | Intercept | 0.02 | 0.004 | 6.19 | <0.001 |
|  |  | Network size | -0.002 | 0.0001 | -15.87 | <0.001 |
|  | ***Proportion*** | Intercept | -0.06 | 0.005 | -12.54 | <0.001 |
|  |  | Network size | 0.001 | 0.0001 | 10.06 | <0.001 |
|  | ***Conformity*** | Intercept | -0.02 | 0.003 | -8.44 | <0.001 |
|  |  | Network size | 0.001 | 0.0001 | 7.70 | <0.001 |
| ***Mean correlation coefficient for weighted betweenness*** | ***Simple*** | Intercept | -0.14 | 0.012 | -11.6 | 0.002 |
|  |  | Network size | -0.001 | 0.0002 | -3.17 | 0.002 |
|  | ***Threshold*** | Intercept | 0.01 | 0.002 | 3.62 | <0.001 |
|  |  | Network size | -0.001 | 0.0001 | -7.32 | <0.001 |
|  | ***Proportion*** | Intercept | -0.05 | 0.004 | -12.55 | <0.001 |
|  |  | Network size | 0.001 | 0.0001 | 9.30 | <0.001 |
|  | ***Conformity*** | Intercept | -0.02 | 0.002 | -8.83 | <0.001 |
|  |  | Network size | 0.001 | 0.0001 | 7.59 | <0.001 |
